# Multi-view based integrative analysis of gene expression data for identifying biomarkers

**DOI:** 10.1038/s41598-019-49967-4

**Published:** 2019-09-18

**Authors:** Zi-Yi Yang, Xiao-Ying Liu, Jun Shu, Hui Zhang, Yan-Qiong Ren, Zong-Ben Xu, Yong Liang

**Affiliations:** 1 Faculty of Information Technology & State Key Laboratory of Quality Research in Chinese Medicines, Macau University of Science and Technology, Taipa, 999078 Macau China; 2 Computer Engineering Technical College, Guangdong Polytechnic of Science and Technology, Zhuhai, 519090 China; 3 School of Mathematics and Statistics & Ministry of Education Key Lab of Intelligent Networks and Network Security, Xi’an Jiaotong University, Xi’an, 710049 China

**Keywords:** Computational models, Data integration, Data mining, Machine learning

## Abstract

The widespread applications in microarray technology have produced the vast quantity of publicly available gene expression datasets. However, analysis of gene expression data using biostatistics and machine learning approaches is a challenging task due to (1) high noise; (2) small sample size with high dimensionality; (3) batch effects and (4) low reproducibility of significant biomarkers. These issues reveal the complexity of gene expression data, thus significantly obstructing microarray technology in clinical applications. The integrative analysis offers an opportunity to address these issues and provides a more comprehensive understanding of the biological systems, but current methods have several limitations. This work leverages state of the art machine learning development for multiple gene expression datasets integration, classification and identification of significant biomarkers. We design a novel integrative framework, MVIAm - Multi-View based Integrative Analysis of microarray data for identifying biomarkers. It applies multiple cross-platform normalization methods to aggregate multiple datasets into a multi-view dataset and utilizes a robust learning mechanism Multi-View Self-Paced Learning (MVSPL) for gene selection in cancer classification problems. We demonstrate the capabilities of MVIAm using simulated data and studies of breast cancer and lung cancer, it can be applied flexibly and is an effective tool for facing the four challenges of gene expression data analysis. Our proposed model makes microarray integrative analysis more systematic and expands its range of applications.

## Introduction

Microarray technology is one of the most recent advances being used for cancer research, which can measure the expression levels of many thousands or tens of thousands of genes simultaneously. With the rapid development of microarray technology, many database repositories of high throughput gene expression data have been created and published for researchers to use, Gene Expression Omnibus (GEO), for example, currently have stored more than 2.76 million samples over 105,000 studies^1^. The use of gene expression datasets to discover highly reliable biomarkers is an important goal in clinical applications. The significant biomarkers can help researchers to detect the disease in individuals, classify the type of disease, predict the response of therapy and so on^2^.

Analysis of gene expression data using biostatistics and machine learning approaches is facing four major challenges: (1) High noise: Random noise and systematic biases exist in gene expression data not only impact the scientific validity and costs of studies but also disrupts accurate prediction of phenotype that may ultimately impact patients^3,4^. (2) Small sample size with high dimensionality: The gene expression dataset generally contains a large number of genes and small size of samples, which called large *p* & small *n* problem^5^. Only a small fraction of genes are closely relevant to the target disease, and most genes are irrelevant^6^. From a machine learning perspective, numerous irrelevant genes may introduce noise and reduce the performance of the classifier^7,8^. (3) Batch effects: It occurs because measurements are affected by many factors including experiments principle, data collection standards, and personnel differences. The systematic noise introduced when samples are processed in multiple batches have a detrimental effect on data derived from microarrays^9,10^. (4) Low reproducibility of significant biomarkers: The published significant biomarkers from internal validation rarely overlap with other research groups^11^. These four issues reveal the complexity of gene expression data, which constrains the development of microarray technology in clinical applications.

To face these challenges and take advantage of multiple published gene expression datasets, the integrative analysis of gene expression data has become an effective tool by aggregating multiple datasets and increasing the statistical power in identifying a small subset of genes to effectively predict the type of the disease^12,13^. Current microarray integrative analysis was first proposed by Hamid *et al*.^14^, basically classified into “late stage” data integration and “early stage” data integration. However, current methods for microarray integrative analysis have several limitations. Most “late stage” data integration methods identify genes based on combining univariate summary statistics, such as *p*-value^15^, effect size^16^ and rank aggregation^12,17^. As a result, it is difficult to identify non-redundant significant genes and systematically determine (e.g. cross-validation) how many genes to include in the subset, such as GeneMeta^18^ and metaArray^19^. Moreover, such methods neglect correlations among genes and do not eliminate the batch effects between different datasets. Current “early stage” data integration methods usually apply one cross-platform normalization method to aggregate multiple datasets into a single unified large dataset. After that, classification and variable selection for the merged dataset can be achieved by the machine learning methods. For example, Ma *et al*.^20^ proposed the meta threshold gradient descent regularization (MTGDR) for gene selection in the integrative analysis of gene expression data. Meta-lasso method was published by Li *et al*.^21^, which not only boosts the statistic power to identify significant genes but also keeps the flexibility of gene selection. Recently, Hughey *et al*.^22^ developed integrative analysis using elastic net penalized with logistic regression model (L_*EN*_), a powerful and versatile method for variable selection in classification. Special emphasis, cross-platform normalization is an essential part of the “early stage” data integration, because it can eliminate the differences between datasets from different microarray platforms while preserving underlying the differences in biology^23^. A number of cross-platform normalization methods have been developed and provide effective batch adjustment for microarray data, such as ComBat^24^, cross-platform normalization (XPN) method^25^, and batch effects removal (ber)^26^. However, different cross-platform normalization methods are based on different statistical models with different accuracy, precision and overall effectiveness^27^. Current “early stage” data integration methods usually apply one cross-platform normalization method, which cannot ensure maximum elimination of the batch effects. Beyond that, none of these integrative analysis methods have a robust learning mechanism to minimize the influence of the noise. Therefore, there is a crucial need for a novel integrative analysis method for robust analysis of the microarray data, prediction of cancer types and identification of significant biomarkers.

We design a novel integrative framework called MVIAm (Multi-View based Integrative Analysis of microarray data for identifying biomarkers). MVIAm can be divided into three phases: pre-processing each dataset, aggregation and generate multi-view data, and analysis of multi-view data. MVIAm aggregates multiple microarray gene expression datasets through different cross-platform normalization methods and generates multiple aggregated gene expression datasets. Each aggregated dataset has the same set of samples and features but is generated by the different statistical models, which belongs to one type of multi-view data^28^. The novel integrative framework MVIAm extends the traditional “early” stage data integration to multi-view data integration. Generally, multi-view data contains complementary information and has more comprehensive information than those of single-view data^29^. In recent years, several multi-view machine learning methods for integrating multi-view data have been developed^28,30^. The supervised multi-view data integration methods generally include concatenation-based and ensemble-based integration^31^. MVIAm enables more multi-view machine learning methods for supervised homogeneous data integration. The multi-view gene expression data generated by MVIAm has the following characteristics:

Multi-view data generated by MVIAm can significantly increase the sample size, which greatly alleviates large *p* & *n* problem and increase the statistical power in identifying biomarkers.

- Multi-view data typically contains complementary information and has more comprehensive understanding of the biological systems.
- The batch effects cannot be completely eliminated, meaning that each view of the data still has different types of bias.

Although quality control and different cross-platform normalization methods are used to process gene expression data, it is inevitable that the data has noises and biases. In the phase of analyzing gene expression data, in order to alleviate the impact of the noise on the learning process and take advantage of significantly increased data, we introduce a robust learning mechanism called self-paced learning^32^. Self-paced learning (SPL) is a typical sample reweighting method, especially used in high noise situations^33^. It was proposed based on the core idea of curriculum learning^34^. Curriculum learning (CL) is inspired by human learning and is learned by gradually including samples from easy to complex into the training process. SPL embeds curriculum design as a regularization term into the learning objective, automatically select samples into training from easy to complex in a purely self-paced way. Due to its generality and generalization, SPL has been widely used in various tasks^35–38^. Moreover, Meng *et al*.^39^ have provided some new theoretical understanding of the SPL scheme, which helps us have a deep insight into it. To analysis multi-view gene expression data, we propose Multi-View Self-Paced Learning (MVSPL), a robust supervised multi-view data integration method. The main idea of MVSPL is to interactively recommend high-confidence samples with smaller loss values and automatically select samples from easy to complex to train the model for each view.

In summary, the main contributions of this work can be summarized as follows:

- We design a novel framework of gene expression data integration called MVIAm, which can generate multi-view gene expression data based on different cross-platform normalization methods. Moreover, we propose a robust learning method MVSPL to analyze multi-view gene expression data for gene selection and cancer classification problem. It is an effective tool to address the challenges of microarray data analysis.
- Experimental results on both simulation and real experiments substantiate the superiority of MVSPL as compared to a sparse logistic regression model with Lasso (L_1_), a sparse logistic regression model with elastic net (L_*EN*_), ensemble-based elastic net (Ensemble_EN) and SPL.
- Our proposed model makes gene expression integrative analysis more systematic and expands the range of applications that an integrative analysis can be used to address.

## Methods

### The MVIAm integrative framework

**Figure 1:**
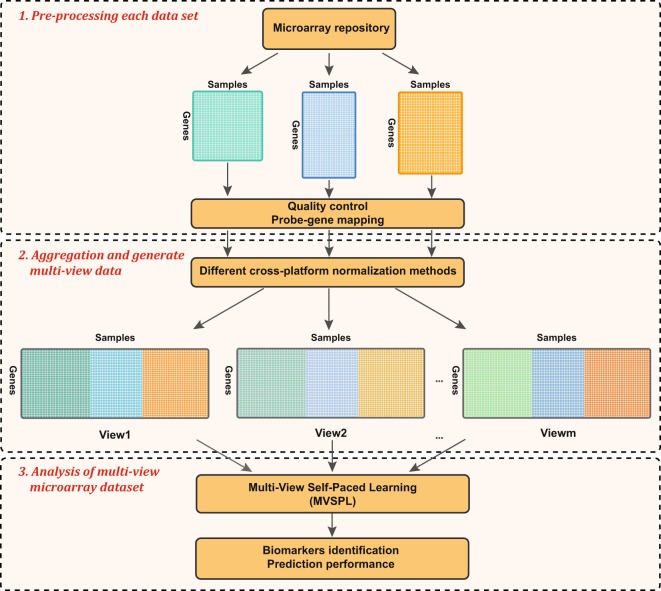
MVIAm, a novel framework for data integrative analysis. The first phase inputs multiple microarray datasets and processes the data according to the pre-processing steps. For the second phase of MVIAm, it applies multiple cross-platform normalization methods to aggregate multiple datasets. Each aggregated dataset possesses the same set of samples and genes, but it is generated by the different statistical normalization models, which belongs to one type of multi-view data. The third phase is the analysis of multi-view microarray data, we propose the MVSPL approach to identify significant biomarkers and predict the type of cancer.

Figure 1 shows the pipeline of the MVIAm, which aggregates multiple microarray datasets and identifies the significant biomarkers, assesses the prediction performance of the model. MVIAm can be divided into three phases: pre-processing each dataset, aggregation and generate multi-view data, and analysis of multi-view data.

#### Pre-processing each data set

The original Affymetrix data was first normalized and log-transformed by a robust multi-array average (RMA)^40^ method. After that, downloading and installing the appropriate custom chip definition files (CDFs) packages according to the type of microarray platform. The CDF package is necessary for probe annotation for Affymetrix data. The probes of the normalized data can be successfully mapped to Entrez Gene IDs by annotation packages in Bioconductor^41^. If multiple probes match a single Entrez ID, we calculated the median of values of those probes as the expression value for this gene.

#### Aggregation and generate multi-view data

One challenge of microarray integrative analysis is that each gene expression dataset may have gene expression values for slightly different sets of genes. Commonly method, the common genes from all gene expression datasets are extracted as the merged set of genes. After that, MVIAm utilizes different cross-platform normalization methods to process the gene expression dataset to eliminate the batch effects. In this work, we use two cross-platform normalization methods to eliminate the batch effects, ComBat^24^ and ber^26^. ComBat is an Empirical Bayes method, includes two methods, a parametric prior method (ComBat_p) and a non-parametric method (ComBat_n), based on the prior distributions of the estimated parameters. Ber, removes batch effects by using a two-stage regression approach, includes two methods, with bagging method (ber_bg) and without bagging method (ber).

#### Multi-view self-paced learning (MVSPL)

Here, we detailed introduce the proposed multi-view self-paced learning (MVSPL) model, which extends the self-paced learning^35^ model to multi-view scenarios. The fundamental concept of SPL please see the part of related work. Suppose given a dataset with multiple views $D=\{({X}_{1}^{(j)},{y}_{1}),({X}_{2}^{(j)},{y}_{2}),\ldots ,({X}_{n}^{(j)},{y}_{n})\}$, where ${X}_{i}^{(j)}=({x}_{i1}^{(j)},{x}_{i2}^{(j)},\ldots ,{x}_{ip}^{(j)})$ is the *i*-th input sample with *p* features under the *j*-th view and *y*_*i*_ is the *i*-th sample with the value 0 or 1 for every view in the classification model. Let $L({y}_{i},f({x}_{i}^{(j)},{\beta }^{(j)}))$ denotes the loss function, which calculates the loss between the real label *y*_*i*_ and the estimated value $f({x}_{i}^{(j)},{\beta }^{(j)})$ in the *j*-th view. The *β*^(*j*)^ represents the model parameter inside the decision function $f({x}_{i}^{(j)},{\beta }^{(j)})$. The objective function of MVSPL can be expressed as:

1 $$\begin{array}{rcl}\mathop{{\rm{\min }}}\limits_{\begin{array}{c}{\beta }^{(j)},{v}^{(j)}\in {[0,1]}^{n},j=1,2,\ldots ,m\end{array}}E({\beta }^{(j)},{v}^{(j)};{\lambda }^{(j)},{\gamma }^{(j)},\delta ) & = & \mathop{\sum }\limits_{j=1}^{m}\mathop{\sum }\limits_{i=1}^{n}{v}_{i}^{(j)}L({y}_{i},{f}^{(j)}({x}_{i}^{(j)},{\beta }^{(j)}))\\  &  & +\,\mathop{\sum }\limits_{j=1}^{m}{\lambda }^{(j)}{\Vert {\beta }^{(j)}\Vert }_{1}-\mathop{\sum }\limits_{j=1}^{m}\mathop{\sum }\limits_{i=1}^{n}{\gamma }^{(j)}{v}_{i}^{(j)}\\  &  & -\delta \sum _{\begin{array}{c}\begin{array}{c}1\le k,j\le m,\\ k\ne j\end{array}\end{array}}{({v}^{(k)})}^{T}{v}^{(j)},\end{array}$$

where *m* denotes the total number of views. ${x}_{i}^{(j)}$ is the *i*-th input sample (*i* = 1, 2, …, *n*) under the *j*-th view, and *y*_*i*_ is the corresponding label of ${x}_{i}^{(j)}$ for every *j*. ${v}_{i}^{(j)}$ denotes the weight of ${x}_{i}^{(j)}$. *λ*^(*j*)^ is a tuning parameter in the *j*-th view, it controls the complexity of the model. *γ*^(*j*)^ denotes the age parameter, which controls the learning pace in each iteration in the *j*-th view. *δ* is the parameter controls influence from other views when one view is going to select more training samples.

MVSPL actually corresponds to the sum of SPL model under multiple views plus a regularization term $${\sum }_{\begin{array}{c}1\le k,j\le m\\ k\ne j\end{array}}{({v}^{(k)})}^{T}{v}^{(j)}$$. This inner product encodes the relationship between multiple views. This new regularizer demonstrates the basic assumption that multi-view data usually contains complementary information and have more comprehensive information than those of single-view data. Therefore, this new regularizer enforces the weight penalizing the loss of one view similar to that of other views.

### The alternative optimization strategy

The alternative optimization strategy (AOS) can be used to solve the MVSPL model. The optimization process is as follows:

#### Initialization

*v*^(1)^, *v*^(2)^, …, *v*^(*m*)^ are zero vectors in *R*^*m*^. *γ*^(1)^, *γ*^(2)^, …, *γ*^(*m*)^ are initialized with small values to allow a few samples into training for the first iteration. *δ* is set as a specific value in the whole learning process. Multiple classifiers are simultaneously trained on all samples in different views to obtain an initial loss of all samples in each view.

#### Update *v*_*i*_^(*k*)^(*k* = 1, 2,…, *m*; *k* ≠ *j*)

The purpose of this step is to prepare confident samples with non-zeros ${v}_{i}^{(k)}$ values for training on the *j*-th view. By calculating the derivative of Eq. (1) with respect to ${v}_{i}^{(k)}$, then we can obtain:

2 $$\begin{array}{l}\frac{\partial E}{\partial {v}_{i}^{(k)}}={L}_{i}({y}_{i},{f}^{(k)}({x}_{i}^{(k)},{\beta }^{(k)}))-{\gamma }^{(k)}-\delta \sum _{\begin{array}{c}1\le j\le m,j\ne k\end{array}}{v}_{i}^{(j)}.\end{array}$$

According to Eq. (2), we can obtain the optimal weight for the *i*-th sample in the *k*-th view:

3 $${v}_{i}^{(k)}=(\begin{array}{ll}1, & {L}_{i}({y}_{i},{f}^{(k)}({x}_{i}^{(k)},{\beta }^{(k)})) < {\gamma }^{(k)}+\delta \sum _{\begin{array}{c}1\le j\le m,j\ne k\end{array}}\,{v}_{i}^{(j)},\\ 0, & otherwise.\end{array}$$

#### Update *v*_*i*_^(*j*)^

This step aims to define which samples will be selected into the training of the *j*-th view. The optimization process for the *v*_*i*_^(*j*)^ is the same as the previous step, expressed as:

4 $${v}_{i}^{(j)}=(\begin{array}{ll}1, & {L}_{i}({y}_{i},{f}^{(j)}({x}_{i}^{(j)},{\beta }^{(j)})) < {\gamma }^{(j)}+\delta \sum _{\begin{array}{c}1\le k\le m,k\ne j\end{array}}{v}_{i}^{(k)},\\ 0, & otherwise.\end{array}$$

The difference is that the samples selected in this step will be directly used for training in the *j*-th view. Furthermore, we can easily observe that samples selected by other views possess higher probabilities than others to be selected into training.

#### Update *β*^(*j*)^

The purpose of this step is to obtain the optimal solution for the *j*-th view. Here, we choose the logistic regression classifier to train the model. Equation (1) degenerates into penalized logistic regression optimization problem:

5 $$\begin{array}{c}{\rm{\min }}\\ {\beta }^{(j)}\end{array}\mathop{\sum }\limits_{i=1}^{n}{v}_{i}^{(j)}{L}_{i}({y}_{i},{f}^{(j)}({x}_{i}^{(j)},{\beta }^{(j)}))+{\lambda }^{(j)}{\Vert {\beta }^{(j)}\Vert }_{1}.$$

This problem can be readily solved by R package glmnet^42^.

Age parameter *γ*^(*j*)^(*j* = 1, 2, …, *m*) is increased to allow more samples with larger loss values into training in the next iteration. When *γ*^(*j*)^ is small, only select easy samples under *j*-th view with small losses. With the growth of the *γ*^(*j*)^, more samples under *j*-th view with larger losses will be gradually selected to train a more “mature” model. Then we repeat the above optimization process with respect to each variable under the different views until the maximum iteration times is reached.

The pipeline of the proposed MVSPL is shown in Supplementary Fig. S1. And the whole process of this alternative optimization strategy for solving MVSPL is summarized in Algorithm 1.

**Algorithm 1:**
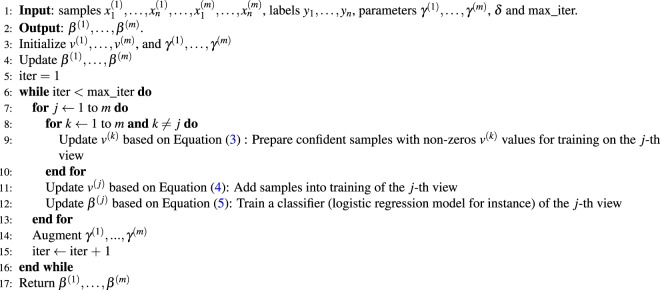
The alternative optimization strategy for solving MVSPL model.

According to Algorithm 1, the MVSPL model can obtain the optimal solution for each view. Algorithm 1 jointly learns the modal parameter *β*^(*j*)^ and the latent weight variables *v*^(*j*)^, where *j* = 1, …, *m*. Steps 7–11 compute the latent weight variables of all samples *n* in multiple views *m* with the time complexity of O(*n* × *m*^2^). With the latent weight variables fixed, Step 12 computes the optimal solution based on the generalized linear model with lasso penalty by using Coordinate Descent algorithm^42^ with the time complexity of O(*n*^2^ × *p*), where *p* represents the number of features and *n* ≪ *p*. This step computes the optimal solution in multiple views, so the time complexity is O(*n*^2^ × *p* × *m*). Due to *m* ≪ *n*, therefore, the time complexity of Algorithm 1 is O(*n*^2^ × *p* × *m*).

In the test phase, when the test dataset *D*^′^ = {*X*_1_, *X*_2_, …, *X*_*u*_} with multiple views (1, 2, …, *m*) are coming, where *u* is the number of test samples. We first fix *β*^(1)^, *β*^(2)^, …, *β*^(*m*)^, and then predict the optimal *y*_*k*_ by solving the following minimization problem:

6 $$\begin{array}{l}{y}_{k}=\mathop{argmin}\limits_{{y}_{k}}\mathop{\sum }\limits_{j=1}^{m}{L}_{k}({y}_{k},{f}^{(j)}({x}_{k}^{(j)},{\beta }^{(j)}))\end{array}$$

### Related work

#### Self-paced learning (SPL)

The self-paced learning model combines a weighted loss term for all samples and a general self-paced regularizer imposed on the samples weight. Suppose given a dataset *D* = {(*X*_1_, *y*_1_), (*X*_2_, *y*_2_), …, (*X*_*n*_, *y*_*n*_)}, where *X*_*i*_ = (*x*_*i*1_, *x*_*i*2_,…, *x*_*ip*_) is the *i*-th input sample with *p* features and *y*_*i*_ is class of the *i*-th sample (e.g. *y*_*i*_ ∈ {0, 1}). Let *L*(*y*_*i*_,*f*(*x*_*i*_, *β*)) denotes the loss function, which calculates the loss between the real label *y*_*i*_ and the estimated value *f*(*x*_*i*_, *β*). The *β* represents the model parameter inside the decision function *f*(*x*_*i*_, *β*). The goal of the SPL is to jointly learn the model parameter *β* and the latent weight variable *v* = [*v*_1_, *v*_2_, …, *v*_*n*_] by minimizing:

7 $$\mathop{{\rm{\min }}}\limits_{\beta ,v\in {[0,1]}^{n}}E(\beta ,v;\lambda ,\gamma )=\mathop{\sum }\limits_{i=1}^{n}{v}_{i}L({y}_{i},f({x}_{i},\beta ))-\gamma \mathop{\sum }\limits_{i=1}^{n}{v}_{i}+\lambda {\Vert \beta \Vert }_{1}$$

where *γ* is the age parameter for controlling the learning pace and *λ* is a tuning parameter. The alternative optimization strategy algorithm can effectively solve the SPL problem. When *β* is fixed, the optimum weight variable ${v}^{\ast }=[{v}_{1}^{\ast },{v}_{2}^{\ast \ast },\mathrm{...},{v}_{n}^{\ast }]$ can be calculated by:

8 $${v}_{i}^{\ast }=\{\begin{array}{ll}1, & L({y}_{i},f({x}_{i},\beta )) < \gamma \\ 0, & {\rm{otherwise}}\end{array}$$

By jointly updating model parameter *β* and the latent weight variable *v*, we can conclude that: (1) When updating *v* with a fixed *β*, if the loss value of a sample is smaller than the age parameter *γ*, then the sample is treated as an easy sample with ${v}_{i}^{\ast }=1$, otherwise, ${v}_{i}^{\ast }=0$. (2) When updating *β* with a fixed *v*, using the selected samples (${v}_{i}^{\ast }=1$) to train the classifier. (3) Before running the next iteration, increase the age parameter *γ* to adjust the learning pace. When *γ* is small, only select easy samples with small loss values. With *γ* increases, more samples with larger losses will be gradually selected to train a more “mature” model.

By jointly learning the model parameter *β* and the latent weight variable *v* based on the iterative algorithm with gradually increasing the age parameter, more samples can be automatically selected into training from easy to complex in a self-paced way.

## Results

We demonstrate the performance of the proposed MVSPL in simulation and real microarray experiments. Four methods are compared with the MVSPL method: Sparse logistic regression with the Lasso penalty (L_1_)^43^, Sparse logistic regression with the elastic net penalty (L_*EN*_)^44^, Ensemble-based elastic net (Ensemble_EN)^45^ and SPL^32^. When MVIAm generates single-view data, it degenerates into traditional “early stage” data integration, and data analysis can be performed by L_1_, L_*EN*_ and SPL. Ensemble_EN constructs a prediction model on each view of data before combing the model predictions and obtains the final prediction result based on Eq. (6).

### Analysis of simulated data

We generate three independent simulated datasets for integration and each dataset with the character of small sample size and high dimensionality. Using the normal distribution to generate *X* = (*X*_1_, *X*_2_, …, *X*_*n*_) with *n* samples and each samples with *p* features, for the *i*-th sample, *X*_*i*_ = (*x*_*i*1_, *x*_*i*2_, …, *x*_*ip*_). After that, the correlation parameter *ρ* can be added to the simulated data^46^.

9 $$\begin{array}{l}{x}_{ij}={z}_{ij}\sqrt{1-\rho }+{z}_{i1}\sqrt{\rho },i \sim (1,\ldots ,n),j \sim (2,\ldots ,p).\end{array}$$

where *z*_*ij*_~_*i*.*i*.*d*._*N*(0, 1). The simulated dataset is generated from the logistic regression model, which can be given as:

10 $$\begin{array}{l}log(\frac{{y}_{i}}{1-{y}_{i}})={\beta }_{0}+\mathop{\sum }\limits_{j=1}^{p}{x}_{ij}{\beta }_{j}+\sigma \cdot \varepsilon ,\end{array}$$

where *ε* = (*ε*_1_, *ε*_2_, …, *ε*_*n*_)^*T*^ is the independent random errors from *N*(0, 1), *σ* is the noise control parameter.

We generated simulated data by the above procedure. Three independent simulated datasets were generated with the same number of variables (*p* = 2000). The coefficient *β* is set as follows:

11 $$\begin{array}{l}\beta =(\mathop{\underbrace{1.5,-\,1.2,1.8,-\,2,2.5,-\,1.2,1,-1.5,2,-\,1.6}}\limits_{10},\mathop{\underbrace{0,\cdots ,0}}\limits_{1990}).\end{array}$$

Four scenarios were designed for the simulated experiment:

**Scenario 1**: The sample size *n*_*dataset*1_ = 100, *n*_*dataset*2_ = 100 and *n*_*dataset*3_ = 100, the correlation coefficient *ρ* = 0, 0.2, 0.4, 0.6 and 0.8, the noise control parameter *σ* = 0.

**Scenario 2**: The sample size *n*_*dataset*1_ = 100, *n*_*dataset*2_ = 100 and *n*_*dataset*3_ = 100, the noise control parameter *σ* = 0, 0.2, 0.4, 0.6 and 0.8, the correlation coefficient *ρ* = 0.

**Scenario 3**: The sample size *n*_*dataset*1_ = 50, *n*_*dataset*2_ = 100 and *n*_*dataset*3_ = 150, the noise control parameter *σ* = 0, 0.4 and 0.8, the correlation coefficient *ρ* = 0.

**Scenario 4**: The sample size *n*_*dataset*1_ = 100, *n*_*dataset*2_ = 100 and *n*_*dataset*3_ = 100, the noise control parameter *σ*_*dataset*1_ = 0.1, *σ*_*dataset*2_ = 0.2 and *σ*_*dataset*3_ = 0.3, the correlation coefficient *ρ* = 0.2.

Three independent simulated datasets are processed based on MVIAm and aggregated into a large multi-view dataset. We use four functions ComBat_p, ComBat_n, ber and ber_bg to eliminate batch effects and generate view1, view2, view3 and view4 of the aggregated multi-view data, respectively. L_1_, L_*EN*_ and SPL achieve the best performance in the view of data by using ComBat_p to eliminate the batch effects. Therefore, these three competing methods use the view1 of the aggregated dataset for data analysis in four scenarios. The proposed MVSPL and Ensemble_EN have the flexibility to analyze data in multiple views. In Scenarios 1, 2 and 3, MVSPL and Ensemble_EN perform data analysis through two views of data: view1 and view2. In Scenario 4, we further explore our proposed method and its flexible scalability. Perform MVSPL through the interaction of two views, three views and four views of data, respectively. In the simulated experiment, we first combine independent simulated datasets into a large aggregated dataset. Then, the aggregated dataset is divided into two groups with random sampling, 70% samples for training and remaining samples for testing. The estimation of the optimal regularization parameter *λ* of the training dataset is obtained by 10-fold cross-validation. We repeat this procedure 30 times and report the average measurement.

To evaluate the prediction performance of classifiers, the accuracy, sensitivity, specificity and AUC are used in the simulation and real experiments. The definitions of these evaluation indicators can refer to^47,48^. In addition, the evaluation indicators for variable selection are defined as follows^49^:

12 $$\begin{array}{rcl}TruePositive(TP) & = & {|\beta .\ast \hat{\beta }|}_{0},TrueNegative(TN)={|\bar{\beta }.\ast \overline{\hat{\beta }}|}_{0}\\ FalsePositive(FP) & = & {|\bar{\beta }.\ast \hat{\beta }|}_{0},FalseNegative(FN)=\,{|\beta .\ast \overline{\hat{\beta }}|}_{0}\\ \beta -sensitivity & = & \frac{TP}{TP+FN},\beta -specificity=\,\frac{TN}{TN+FP}\end{array}$$

where the |·|_0_ represents the number of non-zero elements in a vector. The logical not operators of *β* and $\hat{\beta }$ are $\bar{\beta }$ and $\overline{\hat{\beta }}$, respectively. And.* is the element-wise product.

**Figure 2:**
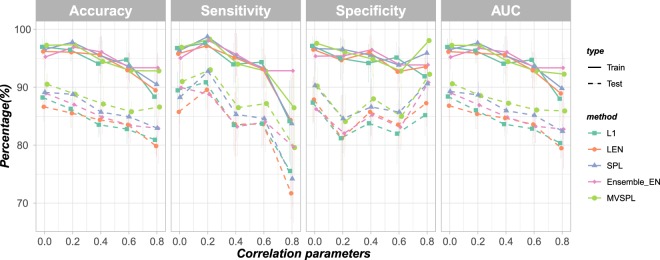
Prediction performance of the different methods with different correlation coefficient parameters. The error bars represent the standard deviation (SD).

In Scenario 1, we explored the effect of different correlation coefficient parameters on the performance of the five methods. As shown in Fig. 2, for the training dataset, the difference in prediction performance of all the methods is quite small. For the test dataset, it can be clearly seen that as the correlation parameter *ρ* increases, the prediction accuracy of all the five methods are decreased, expect for MVSPL in *ρ* = 0.8. The generalization ability of MVSPL and SPL are obviously superior to L_1_, L_*EN*_ and Ensemble_EN. The average test accuracy, sensitivity, and AUC obtained by MVSPL are higher than the other competing methods with varying correlation coefficient parameters *ρ*. The results obtained by SPL are slightly inferior to MVSPL but better than the other three methods in most situations. Moreover, Ensemble_EN outperforms L_1_ and L_*EN*_ with varying correlation parameters.

**Figure 3:**
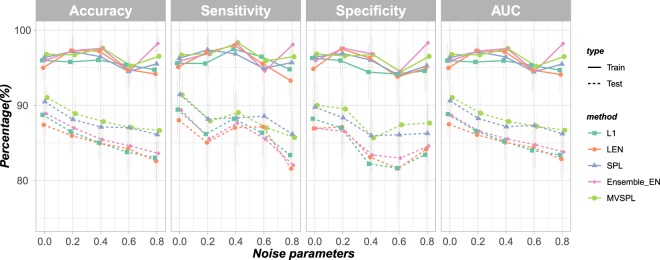
Prediction performance of the different integrative analysis methods with different noise control parameters. The error bars represent the standard deviation (SD).

In Scenario 2, we explored the effect of different noise control parameters on the performance of the five methods. As shown in Fig. 3, consistent with the results of Scenario 1, all methods with the similar prediction performance in the training dataset. For the test dataset, when the noise control parameter increases, the prediction accuracy of all the competing methods are decreased. MVSPL and SPL demonstrate the excellent generalization performance. The average test accuracy and AUC obtained by MVSPL are superior to other competing methods with varying noise control parameters *σ*. For instance, with noise parameter *σ* = 0.4, the average test accuracy of MVSPL is 87.84% superior to 85.04%, 84.96%, 87.11% and 85.44% obtained by L_1_, L_*EN*_, SPL and Ensemble_EN, respectively. In addition, the average test prediction performance of Ensemble_EN performs better than the single-view based methods L_1_ and L_*EN*_ in all cases of Scenario 2.

Table 1 shows the variable selection performance of all the five methods in Scenarios 1 and 2. *β*-sensitivity and *β*-specificity are used to evaluate the variable selection performance. It can be obviously seen that our method achieves the best *β*-sensitivity performance across all cases of simulated experiments. For instance, with noise parameters *σ* = 0.6, the average *β*-sensitivity performance of MVSPL is 91.73% higher than 91.12%, 91.94%, 90.23% and 91.67% obtained by L_1_, L_*EN*_, SPL and Ensemble_EN, respectively. Moreover, by analyzing more views of data, it can improve the *β*-sensitive performance and help identify the significant variables. The average *β*-sensitivity of MVSPL and Ensemble_EN are superior to other single-view analysis methods in most cases. For example, the average *β*-sensitivity of MVSPL and Ensemble_EN are 91.09% and 90.34% better than 88.18%, 88.91% and 88.48% obtained by L_1_, L_*EN*_ and SPL with the noise parameter *σ* = 0.8. The *β*-specificity of all the methods is relatively close in different parameters, between 97.0% to 99%.

**Table 1:** Variable selection performance (%) of the different integrative analysis methods with different parameters.

Method	Correlation coefficient parameters					Noise control parameters				
	*ρ* = 0	*ρ* = 0.2	*ρ* = 0.4	*ρ* = 0.6	*ρ* = 0.8	*σ* = 0	*σ* = 0.2	*σ* = 0.4	*σ* = 0.6	*σ* = 0.8
	***β*** **-sensitivity**									
L_1_	91.21	94.85	88.79	82.24	66.67	90.48	90.82	91.12	90.52	88.18
L_*EN*_	90.91	93.84	88.42	82.33	67.58	90.27	91.24	91.94	89.70	88.91
SPL	90.91	94.67	88.48	83.19	67.64	91.52	92.94	90.23	90.61	88.48
Ensemble_EN	89.67	93.67	92.33	87.67	68.33	89.67	92.33	91.67	90.67	90.34
MVSPL	**92.73**	**95.45**	**92.73**	**88.18**	**69.54**	**92.73**	**93.73**	**92.18**	**91.73**	**91.09**
	***β*** **-specificity**									
L_1_	98.71	98.71	98.79	98.46	98.87	98.81	98.79	98.68	98.56	98.63
L_*EN*_	98.82	98.79	98.51	98.92	98.24	98.98	98.11	98.35	98.75	98.98
SPL	98.71	98.46	98.32	98.72	98.00	98.66	97.96	98.42	98.55	98.62
Ensemble_EN	98.77	98.20	98.01	98.49	97.90	98.54	97.86	97.44	98.55	96.94
MVSPL	98.42	98.44	97.86	98.16	98.37	98.50	97.49	98.02	97.75	97.01

The mean variable selection performance over 30 repetitions of the simulated experiments in Scenarios 1 and 2 are reported, and the best *β* -sensitivity are highlighted in bold.

**Figure 4:**
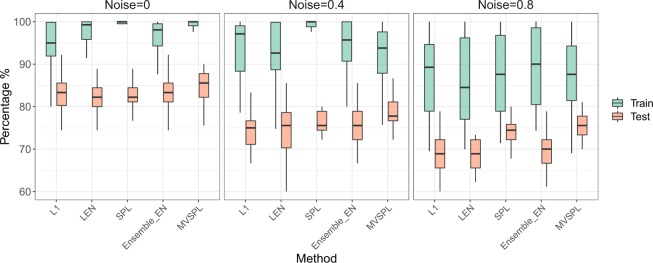
Boxplot diagram of training and test accuracy for the different methods with 30 repetitions in Scenario 3.

In Scenario 3, we explored the effect of different sample sizes on the performance of the five methods. As shown in Fig. 4, we can clearly observe that the test accuracy of MVSPL has achieved the optimal results. MVSPL and SPL exhibit better generalization capabilities compared to other methods, especially in high noise case *σ* = 0.8. Furthermore, the test accuracy of multi-view based method Ensemble_EN is superior to the single-view based methods *L*_1_ and *L*_*EN*_ in Scenario 3.

To further evaluate the performance of the proposed MVSPL method, we designed Scenario 4 in the simulated experiment. The prediction performance of MVSPL in the different number of views is shown in Supplementary Fig. S2. When the number of views increases, the accuracy, sensitivity, specificity and AUC for the test dataset obtained by MVSPL are improved. And we also compare the prediction performance of MVSPL in three views and each of its views. Supplementary Fig. S3 clearly shows that the prediction performance in each single views of MVSPL is worse than that of MVSPL in all views.

To sum up, according to the results of simulated experiments, we can conclude that:

- MVSPL achieves the best generalization ability than the competing methods. The performance of MVSPL outperforms other competing methods with varying correlation parameters and noise parameters.
- By analyzing more views of data, it possible to improve the prediction and variable selection performance. The average performance of MVSPL and Ensemble_EN are superior to the corresponding single-view based methods in most cases.
- When the number of views increases, the prediction performance of MVSPL are improved. This implies that batch effects have an effect for data analysis and more views will contain more comprehensive information.

### Real microarray datasets

We curated data from eight publicly available microarray studies, four breast cancer datasets (same platform) and four lung cancer datasets (disparate platform) (Tables 2 and 3). All of these four breast datasets were produced by the same microarray platform HG-U133A. Classification of breast cancer samples aims to distinguish between the sample’s estrogen receptor (ER) status (+ve or −ve). Four publicly available lung cancer microarray datasets come from disparate platforms. All these publicly available cancer gene expression datasets can be download from GEO (https://www.ncbi.nlm.nih.gov/geo/).

**Table 2:** Four publicly available breast cancer gene expression datasets used in the real data experiments.

Dataset	No. of Probes	Classes (Class1/Class2)	No. of Classes (Class1/Class2)	Affymetrix Platform
GSE1561	22215	−ve/+ve	49 (22/27)	HG-U133A
GSE6532	22283	−ve/+ve	125 (40/85)	HG-U133A
GSE20437	22283	−ve/+ve	18 (9/9)	HG-U133A
GSE22093	22283	−ve/+ve	82 (41/41)	HG-U133A

**Table 3:** Four publicly available lung cancer gene expression datasets used in the real data experiments.

Dataset	No. of Probes	Classes (Class1/Class2)	No. of Classes (Class1/Class2)	Affymetrix Platform
GSE10072	22284	Normal/Tumor	107 (49/58)	U133A
GSE19188	54675	Normal/Tumor	179 (88/91)	U133 Plus 2.0
GSE19804	54676	Normal/Tumor	120 (60/60)	U133 Plus 2.0
GSE43346	22283	Normal/Tumor	65 (42/23)	U133A

### Analysis of real data

For the real microarray data, two types of experimental designs are used in this work. One type evaluates the performance using a random partition. The other type validates the prediction performance on the independent datasets. All publicly available cancer datasets are processed and aggregated in the manner described above (Supplementary Tables S1 and S2). All of publicly available gene expression datasets used in this paper have the class information. Special note, L_1_, L_*EN*_ and SPL achieve the best performance in the view of data by using ComBat_p to eliminate the batch effects. Therefore, these three methods use this view of the aggregated dataset for data analysis in real data analysis. MVSPL and Ensemble_EN analyze two views of data in the real data experiments, which use ComBat_p and ComBat_n to eliminate the batch effects.

#### Evaluating the performance using a random partition

For the part of evaluating the performance using a random partition, we randomly divide the datasets such that 70% of the datasets become the training samples and the remaining samples become the test samples. The estimation of the optimal regularization parameter *λ* of the training dataset is obtained by 10-fold cross-validation. We repeat this procedure 30 times and report the average measurement and standard error.

**Figure 5:**
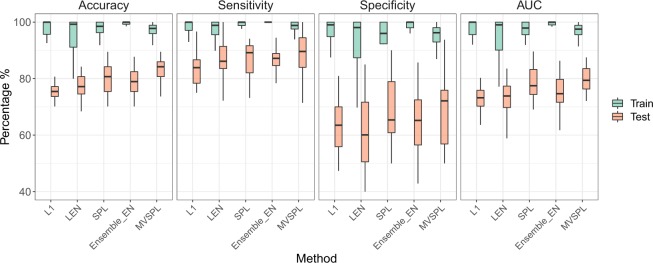
Boxplot diagram of training and test prediction performance for the methods with 30 repetitions in breast cancer dataset.

**Figure 6:**
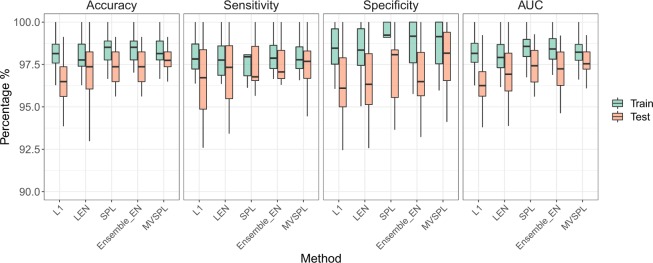
Boxplot diagram of training and test prediction performance for the methods with 30 repetitions in lung cancer dataset.

Figures 5 and 6 plot the box plot analysis of training and test prediction performance calculated on breast and lung cancer datasets under 30 repetitions, respectively. As shown in Fig. 5, for the training dataset, all the five methods achieve desirable performance. For instance, the median average training accuracy of all methods have obtained more than 94%. For the test dataset, the proposed MVSPL has the superior performance compared to other competing methods. For example, the median test accuracy of MVSPL is 84.21%, which is obviously better than 75.44%, 77.19%, 80.70% and 76.90% obtained by L_1_, L_*EN*_, SPL and Ensemble_EN, respectively. Our method achieves the best generalization ability than the competing methods. For lung cancer dataset, as shown in Fig. 6, the training and test prediction performance of all the five methods have reached more than 90%. Our proposed MVSPL method still obtains better classification accuracy, sensitivity, specificity and AUC than other methods. The average number of selected genes for all methods is summarized in Supplementary Table S3.

#### Validating the classifier on independent dataset

For the part of validating the classifier on independent dataset, the design of the validation process is the same as that of metAnalyzeAll^22^. After pre-processing each dataset individually, all the training datasets and the independent validation dataset are merged in the manner described above. The classifier is trained on the samples from the aggregated training dataset and the optimal regularization parameter *λ* is obtained by 10-fold cross-validation. After that, the classifier is tested on the samples from the independent validation dataset.

**Figure 7:**
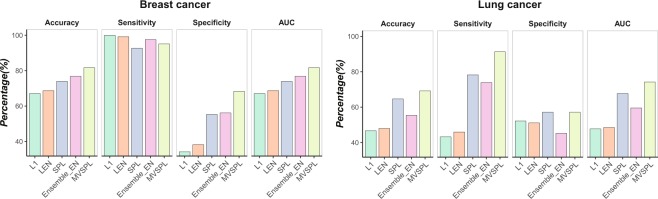
Validation performance comparisons of different integrative analysis methods in the validation datasets of breast cancer and lung cancer studies.

Figure 7 compares the validation prediction performance of L_1_, L_*EN*_, SPL, Ensemble_EN and MVSPL in the validation datasets of breast cancer and lung cancer studies. Validating classifiers on the validation dataset, MVSPL consistently outperforms other competing methods in cancer classification problem. As shown in the left hand of Fig. 7, in breast cancer study, the validation accuracy, specificity, and AUC of MVSPL is superior to other competing methods, except for sensitivity. Specially, MVSPL achieves approximate 10% validation accuracy gain compared with L_1_ and L_*EN*_. Beyond that, Ensemble_EN with the suboptimal performance. In breast cancer study, multi-view analysis method performs better validation prediction performance than single-view analysis method. For lung cancer study, as shown in the right hand of Fig. 7, the validation prediction performance of the proposed MVSPL method has a significant improvement compared to other methods. For example, the validation sensitivity of MVSPL is 91.30%, which is superior to 43.24%, 45.95%, 78.26% and 73.91% obtained by L_1_, L_*EN*_, SPL and Ensemble_EN, respectively. The validation prediction performance of SPL is inferior to MVSPL but is obviously superior to L_1_, L_*EN*_ and Ensemble_EN. Moreover, the validation results of Ensemble_EN is outperformed than L_1_ and L_*EN*_. To summary, by learning from easy to complex samples and interact with multiple views, MVSPL with the best generalization ability than other competing methods. Generally speaking, MVSPL can be successfully applied to the microarray integrative analysis in cancer classification. The average number of selected genes for all methods is summarized in Supplementary Table S4.

**Figure 8:**
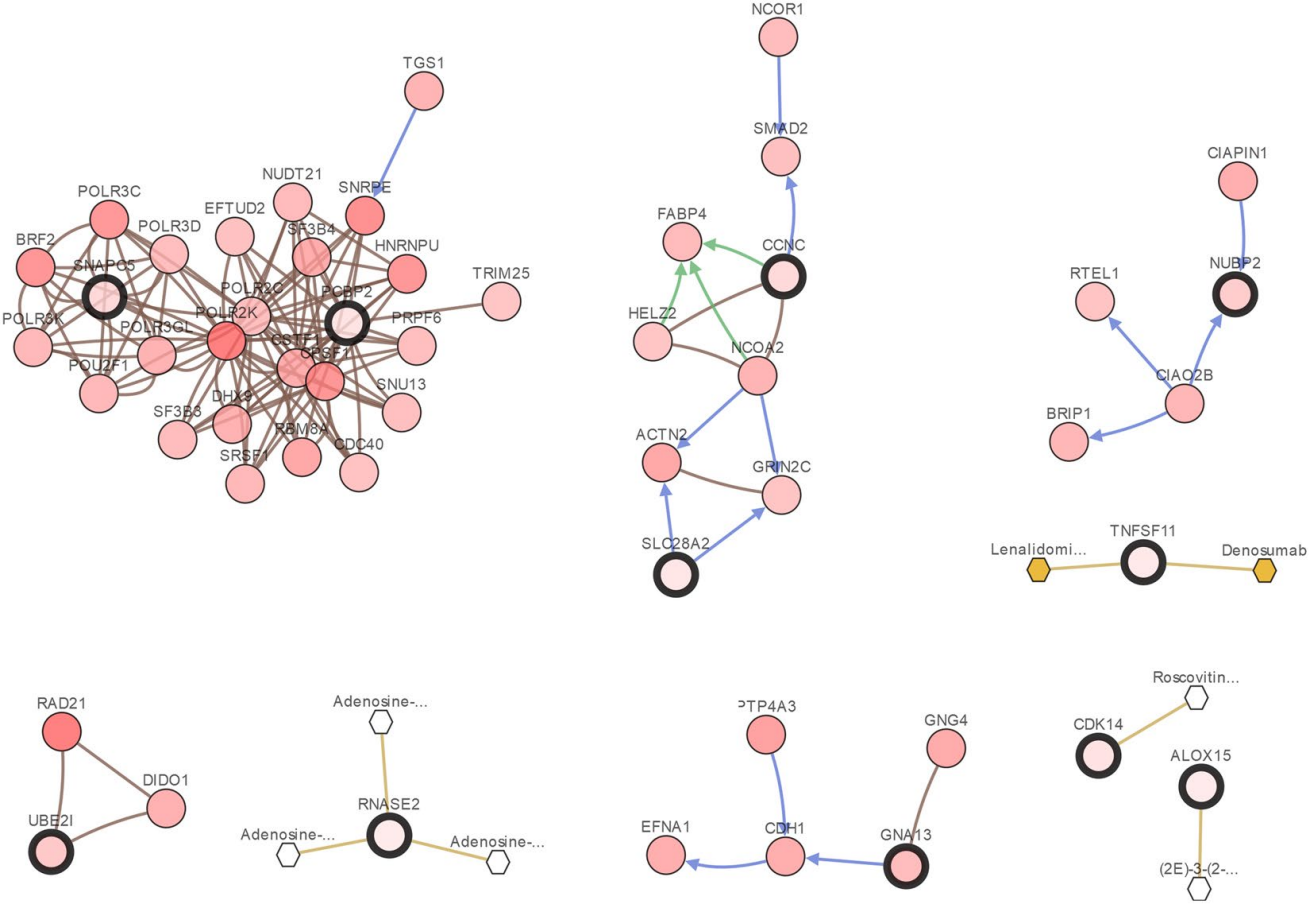
Integrative network view of the genes selected from MVSPL in breast cancer study. The genes corresponding to the selected features are highlighted by a thicker black outline. The rest of the nodes correspond to the genes that are frequently altered and are known to interact with the highlighted genes (based on publicly available interaction data). The nodes are gradient color-coded according to the alteration frequency based on microarray data derived from the TCGA breast cancer dataset via cBioPortal.

**Figure 9:**
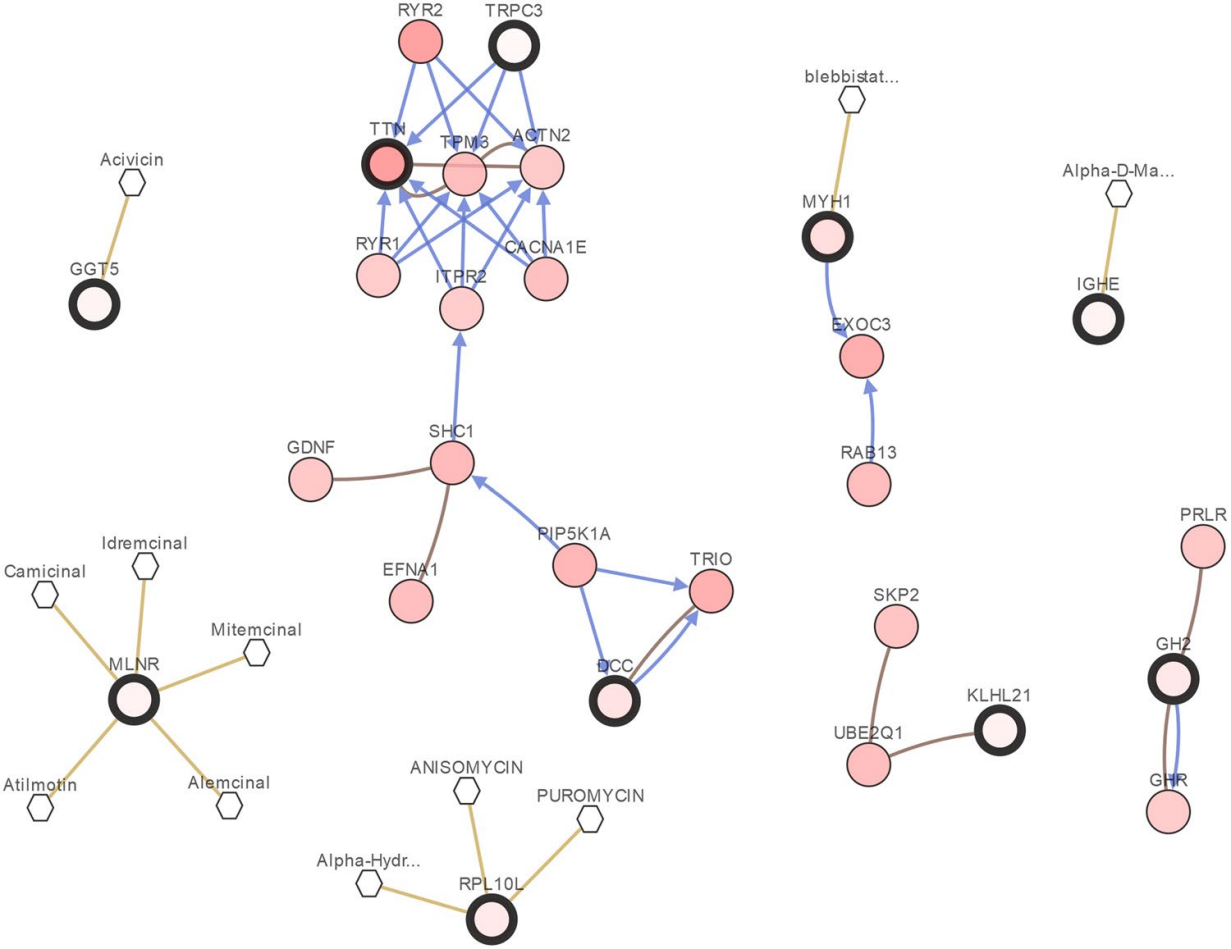
Integrative network view of the genes selected from MVSPL in lung cancer study.

For a brief biological analysis of selected genes, we summaries of the 20 top-ranked genes selected by the five integrative analysis methods in two cancer studies, which are shown in Tables 4 and 5, respectively. To make it easier to demonstrate the interplay between the top selected genes from the microarray integrative analysis, we constructed an network of interactions among the genes using the cBioPortal^50,51^. Figure 8 shows the interactive network of the 20 top-ranked genes selected by MVSPL in breast cancer study. The interactive network shows that SNAPC5, PCBP2 and GNA13 are connected to other frequently altered genes from the TCGA breast invasive carcinoma dataset, which are also selected by other competing methods. Moreover, TNFSF11 is targeted by two FDA approved cancer drugs, it is selected only by MVSPL and SPL. For the genes that are only selected by MVSPL, UBE21 is connected to other frequently altered genes and RNASE2 is targeted by three cancer drugs. For lung cancer study, Fig. 9 shows the interactive network of the 20 top-ranked genes obtained by the proposed MVSPL in lung cancer study. Examination of the resulting network, Fig. 9 shows that TRPC3, DCC, MYH1, GH2 and KLHL21 are linked to other frequently altered genes from the TCGA lung adenocarcinoma dataset. MYH1 and GGT5 are targeted by certain cancer drugs. Moreover, MLNR, IGHE and RPL10L are only obtained by MVSPL, these genes are targets for cancer drugs.

**Table 4:** Top 20 genes selected from different integrative analysis methods in breast cancer dataset.

L_1_	L_*EN*_	SPL	Ensemble_EN		MVSPL	
			View1	View2	View1	View2
**SNAPC5**	**SNAPC5**	RPL7P25	GNL3LP1	**SNAPC5**	CASP5*	CASP5*
KPNA5	RHCG	**CDK14**	SNAPC5	GNL3LP1	**ALOX15**	GFI1B*
RHCG	**ALOX15**	CCNC	XYLB	XYLB	**SNAPC5**	**ALOX15**
**ALOX15**	**CDK14**	RHCG	UTRN	APOBEC1	SLC28A2*	**CDK14**
ANXA2P3	KPNA5	ANXA2P3	SMG8	**CDK14**	**CDK14**	UPK3A
**CDK14**	SMG8	MRM2	ACO1	UTRN	GFI1B*	SLC28A2*
CCNC	AHCYL1	POLR2G	AHCYL1	**RPL7P25**	UPK3A	TNFSF11
**PCBP2**	**PCBP2**	**GNA13**	**CDK14**	APOO	TNFSF11	RNASE2*
AHCYL1	APOO	UPK3A	APOBEC1	RHCG	CCNC	CCNC
SMG8	CCNC	**ALOX15**	**ALOX15**	AHCYL1	UBE2I*	**SNAPC5**
APOO	**GNA13**	RBBP9	SLC25A31	RIMS2	MPZL2*	**PCBP2**
ANAPC10	ANXA2P3	HIGD1B	SERPINB8	SMG8	**PCBP2**	FGGY*
SRD5A2	UTRN	NUBP2	APOO	ACO1	SETX*	MAT2A*
**RPL7P25**	MRM2	SMG8	KPNA5	**PCBP2**	NNAT*	**RPL7P25**
MRM2	ANAPC10	**SNAPC5**	RIMS2	SERPINB8	IRGQ*	NNAT*
**GNA13**	TRIM13	UBA5	**GNA13**	AKTIP	**GNA13**	ALDH1L1*
COX7BP1	ACO1	TNFSF11	AFDN	FA2H	RNASE2*	SENP6
NUBP2	**RPL7P25**	AKTIP	**PCBP2**	LIPC	NUBP2	IRGQ*
TRIM13	RIMS2	WWOX	RHCG	**ALOX15**	RETREG3*	SETX*
UTRN	BANP	**PCBP2**	WWOX	FOXK2	MAT2A*	FOXK2

^1^The genes with star (*) are the unique gene selected by MVSPL, and the common genes selected by each method are emphasized with bold.

**Table 5:** Top 20 genes selected from different integrative analysis methods in lung cancer dataset.

L1	LEN	SPL	Ensemble_EN		MVSPL	
			View1	View2	View1	View2
**HTN3**	**HTN3**	**HTN3**	**HTN3**	**MYH1**	OR1G1	**HTN3**
**MYH1**	**MYH1**	**MYH1**	**MYH1**	**HTN3**	**HTN3**	OR1G1
**DCC**	**DCC**	**DCC**	**DCC**	**DCC**	**MYH1**	**GH2**
RBM15B	**TRBV10-2**	**TRBV10-2**	**TRBV10-2**	RBM15B	**GH2**	**MYH1**
**TRBV10-2**	**TRPC3**	**GH2**	**TRPC3**	**TRBV10-2**	MASP1	MLNR*
**TRPC3**	RBM15B	NEUROG1	**GH2**	NEUROG1	**TRBV10-2**	**TRBV10-2**
**KLHL21**	**GH2**	PITPNA	RBM15B	OR1G1	**ZNF107**	**ZNF107**
TRAM2	NEUROG1	**TRPC3**	NEUROG1	**TRPC3**	**TRPC3**	**DCC**
NEUROG1	TRAM2	OR1G1	PITPNA	**KLHL21**	MLNR*	**PHEX**
**GGT5**	**GGT5**	RBM15B	TRAM2	EXD3	**GGT5**	IGHE*
**GH2**	PITPNA	MASP1	**GGT5**	**GGT5**	**KLHL21**	FAM120C*
EXD3	EXD3	TRAM2	EXD3	TRAM2	ZNF254*	KLHL21
TTN	**ZNF107**	**GGT5**	**ZNF107**	CARHSP1	**PHEX**	**GGT5**
**ZNF107**	**KLHL21**	OR12D3	**KLHL21**	PITPNA	**DCC**	FAF2*
CARHSP1	TTN	EXD3	OR1G1	**GH2**	TMX2	ADAM3A*
PITPNA	OR12D3	**ZNF107**	TTN	TMX2	CARHSP1	BRD7P3*
MFSD11	OR1G1	**PHEX**	OR12D3	**PHEX**	TTN	MFSD11
**PHEX**	**PHEX**	TTN	**PHEX**	**ZNF107**	MFSD11	**TRPC3**
TMX2	MFSD11	**KLHL21**	MFSD11	MFSD11	IGHE*	AMELX*
LRCH1	CARHSP1	CAMSAP1	CARHSP1	TTN	RPL10L*	MASP1

^1^The genes with star (*) are the unique gene selected by MVSPL, and the common genes selected by each method are emphasized with bold.

In addition, a number of genes selected by the five methods have been reported in the literature. For example, in breast cancer, downregulation of ALOX15 expression has been reported in^52,53^. The upregulated expression of CDK14 promotes tumor cell proliferation, migration and invasion through Wnt/*β*— catenin signaling pathway in breast cancer^54^. UPK3A is highly expressed in breast cancer^55^, which is selected only by MVSPL and SPL. Beyond that, MVSPL selects some other unique genes compared with other methods. Phuong *et al*.^56^ confirmed that MAT2A expression in TAM-resistant human breast cancer tissues was higher than that in TAM-responsive cases. Nass *et al*.^57^ proposed that NNAT expression determined by immunohistochemistry might therefore become a helpful additional biomarker to identify high-risk breast cancer patients. For lung cancer, Greenman *et al*.^58^ reported in 2005 that the role of TTN as a cancer gene is currently a mathematically based prediction and will require direct biological evaluation. And after a few years, Tan H *et al*.^59^ said TTN and/or MUC16 were retained in the top 10 for lung cancer, suggesting their tumorigenic relevance to these cancers. MASP1 is over expressed in lung cancer^60^. In this part, we analysis the 20 top-ranked genes selected by the five methods in two cancer studies in gene level. According to the network of interactions among the genes, we find a few numbers of genes are connected to other frequently altered genes from the publicly available datasets and some genes are targeted by certain cancer drugs.

## Conclusion

Due to the complexity of gene expression data, there are four major issues constrain the development of microarray technology in clinical applications: high noise, large *p* & small *n* problem, batch effects and low reproducibility of significant biomarkers. In this work, we design a novel framework called MVIAm to strive to tackle these issues. MVIAm utilizes different cross-platform normalization methods to minimize the impact of batch effects, keeps as much useful information as possible in the microarray gene expression data. In addition, the aggregated gene expression datasets generated by MVIAm belong to multi-view data. It implies that MVIAm can significantly alleviate the large *p* & small *n* problem compared to the existing integrative analysis methods. Therefore, MVIAm can increase the statistical power in identifying the significant biomarkers. To analysis of multi-view gene expression data, we propose a robust learning mechanism called MVSPL to minimize high noise interference. The MVSPL method can improve the generalization performance by learning multi-view data in a meaningful order and improve the prediction performance by the interaction between multiple views. MVSPL actually corresponds to the sum of SPL model under multiple views plus a regularization term. This method implements robust learning regimes in multiple views under the regularization that the robust loss forms in multiple views are closely related. According to the results of simulation and real data experiments, MVSPL has the superior performance compared with L_1_, L_*EN*_, SPL and Ensemble_EN. Especially in the test and validation dataset, MVSPL shows prominent generalization performance. In a word, MVSPL is a feasible and effective method for variable selection and classification in high dimensional data.

There are some ongoing challenges and promising directions that motivate future work. First, our proposed method conducts variable selection with aggregated microarray data in an “all-in-or-all-out” fashion, that is, a gene identified in all of studies or not identified in any study. However, due to data heterogeneity, there may be some genes are important in some studies while unimportant in others. In the future, we will take this situation into account to improve our model. Second, rapid advances in technology have led to a vast quantity of large-scale molecular omics datasets, it provides a distinct view of the complex biological system. Multi-omics dataset with the same set of samples but several distinct feature sets, which naturally belongs to multi-view data. In the future, we will apply our method to the analysis of multi-omics data. We think the computational analysis of the multi-omics data provides an unprecedented opportunity to deepen our understanding of complex cancer mechanisms. Our proposed method makes integrative analysis more systematic and expands its range of applications.

## Supplementary information

Supplementary

## Data Availability

The code of this paper can be download from https://github.com/must-bio-team/MVIAm.
