# Supplementary material for: Multi-view based integrative analysis of gene expression data for identifying biomarkers
Source: Sci Rep. 2019 Sep 18;9:13504. doi: 10.1038/s41598-019-49967-4 (PMC6751173; doi:10.1038/s41598-019-49967-4)
Supplement: Supplementary file 1 — Supplementary [file 41598_2019_49967_MOESM1_ESM.pdf]

Multi-view based integrative analysis of gene expression data for identifying biomarkers

Zi-Yi Yang[1], Xiao-Ying Liu[2], Jun Shu[3], Hui Zhang[1], Yan-Qiong Ren[1], Zong-Ben Xu[3], and Yong Liang[1,*]

[1]Faculty of Information Technology & State Key Laboratory of Quality Research in Chinese Medicines, Macau University of Science and Technology, Taipa, 999078, Macau
[2]Computer Engineering Technical College, Guangdong Polytechnic of Science and Technology, Zhuhai, 519090, China
[3]School of Mathematics and Statistics & Ministry of Education Key Lab of Intelligent Networks and Network Security, Xi'an Jiaotong University, Xi'an, 710049, China

[*]E-mail: yliang@must.edu.mo

Supplemental Figures and Tables

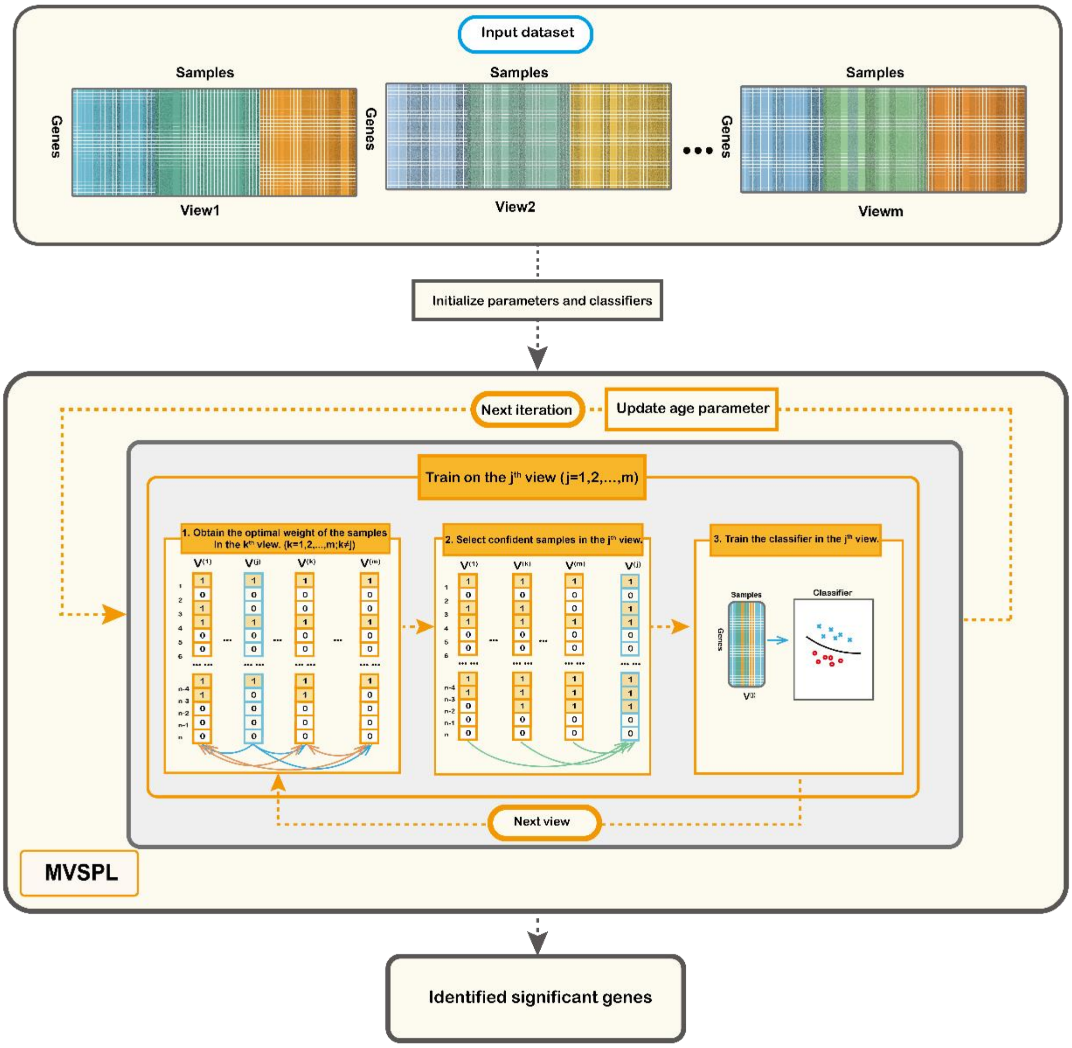

Supplementary Figure S1| The pipeline of the proposed Multi-View Self-Paced Learning (MVSPL). Multi-view gene expression data generated by MVIAm, a bright grid represents a high-quality sample and a darker grid represents a low-quality sample. Initializing the parameters and update estimate coefficients of each view. Before selecting the confident samples of the current trained view, MVSPL first obtains the samples with non-zeros weight variable values for each of the other views. After that, MVSPL selects the confident samples into the training of the classifier in the current view. Age parameter is increased to allow more samples with lower quality into training in the next iteration. Finally, MVSPL identifies significant biomarkers from multi-view gene expression data and predicts the type of the disease.

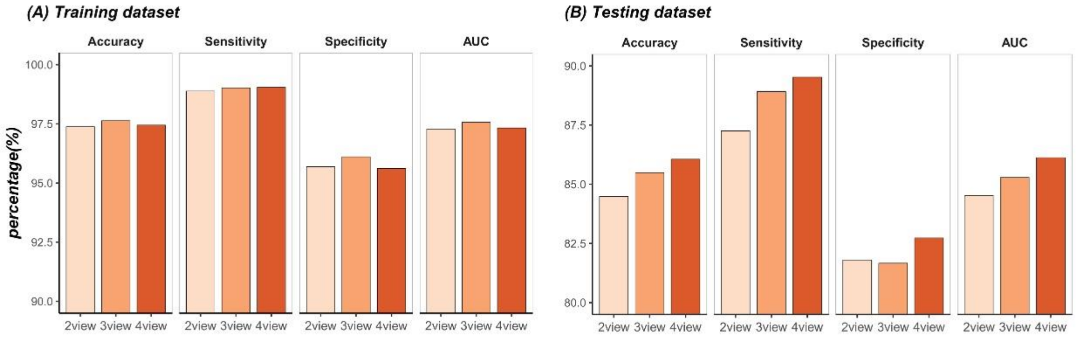

Supplementary Figure S2| Prediction performance of MVSPL in the different number of views.

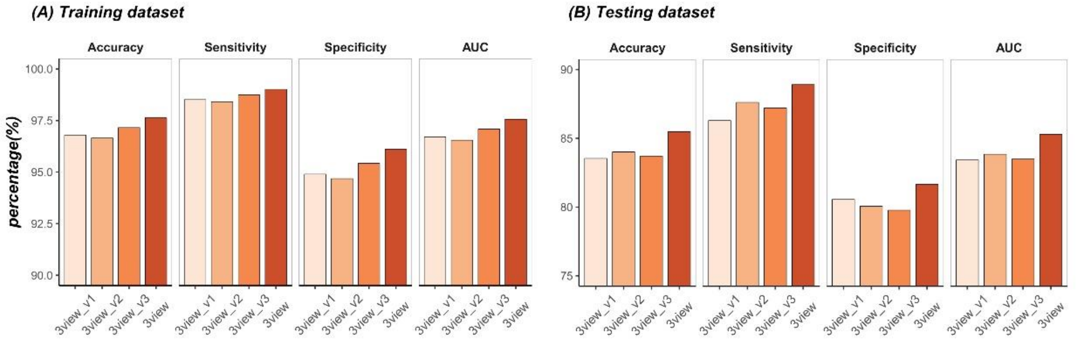

Supplementary Figure S3| Comparisons of the prediction performance of MVSPL (3view) and each of its views.

Supplementary Table S1| The information of the datasets after combination and cross-platform normalization in the random partition real experiment.

Study	Datasets	No. of Samples (Training / Test)	No. of genes
Breast cancer	GSE1561<br>GSE6532<br>GSE20437	192 (134/58)	12320
Lung cancer	GSE10072<br>GSE19188<br>GSE19804	383 (268/115)	12079

Supplementary Table S2| The information of the training dataset and validation dataset after combination and cross-platform normalization.

Study	Training Datasets	Validation Datasets	No. of Samples (Training / Validation)	No. of genes
Breast cancer	GSE1561<br>GSE6532<br>GSE20437	GSE22093	274 (192/82)	12320
Lung cancer	GSE10072<br>GSE19188<br>GSE19804	GSE43346	448 (383/65)	12079

Supplementary Table S3| The average number of selected genes for all competing methods in the part of evaluating the performance using a random partition.

Study	L_1	L_{EN}	SPL	Ensemble_EN	MVSPL
Breast cancer	50.22	52.57	43.87	82.60	76.86
Lung cancer	28.03	33.00	33.30	48.13	49.41

Supplementary Table S4| The number of selected genes for all competing methods in the part of Validating the classifier on independent dataset.

Study	L_1	L_{EN}	SPL	Ensemble_EN	MVSPL
Breast cancer	49	60	62	121	72
Lung cancer	102	130	140	140	201
